# Magnetic resonance elastography in staging liver fibrosis in non-alcoholic fatty liver disease: a pooled analysis of the diagnostic accuracy

**DOI:** 10.1186/s12876-020-01234-x

**Published:** 2020-04-06

**Authors:** Yuanzi Liang, Daowei Li

**Affiliations:** Department of Radiology, The People’s Hospital of China Medical University & The People’s Hospital of Liaoning Province, No. 33, Wenyi Road, Shenhe District, Shenyang, 110016 China

**Keywords:** Magnetic resonance elastography, Non-alcoholic fatty liver disease, Liver fibrosis, Systematic review

## Abstract

**Background:**

This study was performed to systematically evaluate the accuracy of magnetic resonance elastography (MRE) in staging of liver fibrosis in non-alcoholic fatty liver disease (NAFLD).

**Methods:**

PUBMED, EMBASE, Web of Science, CNKI, Cochrane Library database were searched from January 2008 to December 2018 for studies related to MRE in the diagnosis of NAFLD liver fibrosis. The quality of the included literature was assessed by Quality Assessment of Diagnostic Accuracy Studies (QUADAS-2) tool. The pooled sensitivity, the pooled specificity, and area under the receiver operating characteristic curve (AUROC) value was performed by STATA 14.0 software.

**Results:**

A total of 12 studies were included, involving 910 patients. The pooled sensitivity and specificity of each group were 0.77 (95%CI 0.69–0.83) and 0.90 (95%CI 0.83–0.94) for F ≥ 1 (mild liver fibrosis), 0.87 (95%CI 0.74–0.94) and 0.86 (95%CI 0.71–0.94) for F ≥ 2 (significant liver fibrosis), 0.89 (95%CI 0.81–0.94) and 0.84 (95%CI 0.63–0.94) for F ≥ 3(severe liver fibrosis), 0.94 (95%CI 0.85–0.98) and 0.75 (95%CI 0.35–0.94) for F ≥ 4 (early cirrhosis), respectively. The area under the summary receiver operating characteristic (SROC) curve was 0.89, 0.93, 0.93, and 0.95, respectively.

**Conclusions:**

MRE has high accuracy in the diagnosis of hepatic fibrosis staging in patients with NAFLD.

## Key points

1. A total of 910 patients with 12 high-quality studies were systematically analyzed to investigate the accuracy of MRE in staging diagnosis of NAFLD liver fibrosis.
2. The pooled values of sensitivity, specificity, positive likelihood ratio (+LR), negative likelihood ratio (−LR) and diagnostic odds ratio (DOR) were analyzed.
3. The pooled sensitivity and specificity of four stages of liver fibrosis were shown that MRE has high accuracy in the diagnosis of hepatic fibrosis staging in patients with NAFLD.

## Background

Nonalcoholic fatty liver disease (NAFLD), a worldwide epidemic, is a chronic liver disease associated with cirrhosis, which affects 25% of adults. NAFLD is correlated with components of the metabolic syndrome, such as obesity, hypertriglyceridemia, and type 2 diabetes mellitus [1]. NAFLD is classified into simple fatty liver (SFL), nonalcoholic fatty hepatitis (NASH) and NAFLD-related cirrhosis, and NASH is a progressive form that may lead to cirrhosis or hepatocellular carcinoma (HCC) [2, 3]. Due to that staging of fibrosis could indicate disease progression and prognosis in patients with NAFLD, and it is a critical predictors of cirrhosis, hepatocellular carcinoma, and death [4, 5]. Hence, it is critically important for accurate objective tests to detect liver fibrosis in patients with NAFLD.

Biopsy is still considered the gold standard for the diagnosis of liver fibrosis stage in NAFLD [4], but invasive examination may cause sampling error and intra- and inter-observer variability, and may be complicated by morbidity and even death [6]. Therefore, it is needed to develop noninvasive tests that can detect advanced fibrosis in NAFLD patients, but there still remains no noninvasive test approved to diagnose fibrosis in NAFLD patients.

Currently, Noninvasive markers such as Cytokeratin-18, NAFLD fibrosis score, and Enhanced Liver Fibrosis (ELFTM) Test have been proposed for evaluating liver fibrosis, but may not be sufficiently accurate in routine clinical use. Ultrasound-based imaging tests, such as transient elastography (FibroScan) and acoustic radiation force impulse imaging (ARFI) elastography [7] have high (21–50%) failure rates in obese patients [8], and are evaluated only a limited portion of the liver, and findings may be influenced by necroinflammatory activity [7].

Magnetic resonance elastography (MRE), a magnetic resonance-based imaging technique, could utilize shear waves to characterize liver fibrosis. MRE has made significant progress as a non-invasive test for staging liver fibrosis in NAFLD due to its high accuracy in the evaluation of liver fibrosis and also due to the possibility of evaluating a large area of the parenchyma with the option of choosing the region of interest [9]. Although several recent studies have reported a high diagnostic accuracy of MRE in patients with NAFLD [3, 10–20], but those studies had a limited sample size. In this study, we searched all related studies, and performed a systematic review to systematically evaluate the accuracy of MRE in the diagnosis of liver fibrosis in patients with NAFLD.

## Methods

### Literature search strategy

PubMed, Web of Science, CNKI, Embase and Cochrane library database were searched for related literatures in Chinese or English regarding the diagnosis and staging liver fibrosis of NAFLD by MRE. The publication time was from January 2008 to December 2018. The retrieval strategy was (“NAFLD” OR “nonalcoholic fatty liver disease) AND (“liver fibrosis” OR “hepatic fibrosis”) AND (“MRE” OR “MR elastography” OR “magnetic resonance elastography”). In order to perform comprehensive search, the reference lists of the eligible literatures were also searched.

### Inclusion and exclusion criteria

#### Inclusion criteria

1. NAFLD Patient is older than 18 years old;
2. Studies evaluated the diagnostic performance of MRE, if the study includes other diagnostic test, the corresponding data will still be included in the study;
3. Studies used biopsy as the gold standard.
4. If the study population contained NAFLD and other chronic liver disease, the data of NAFLD is separately extracted;
5. Literatures published from January 2008 to December 2018;
6. True positives, false positives, false negatives and true negatives can be directly or indirectly extracted from the literature to construct a 2 × 2 table.

#### Exclusion criteria

1. Reviews, conferences, case reports, animal experiments and technical literature, etc.;
2. The original data is incomplete to construct the four-grid table;
3. Repeated publication.

### Data extraction

Two researchers independently screened the literature and extracted the information according to the established inclusion and exclusion criteria, and cross-checked. If there is any inconsistency, it will be resolved through negotiation. The extracted information mainly includes: the first author, publication year, country, study type, the interval between gold standard and MRE examination, patient information (age, BMI, gender), and four-grid data true positive (TP), false positive (FP), false negative (FN), and true negative (TN).

### Assessment of methodological quality

The quality of the included literature was assessed independently by 2 researchers, and differences were resolved through discussion. Quality Assessment of Diagnostic Accuracy Studies (QUADAS-2) tool was introduced to assess the quality of the included studies. The software Review Manager (version 5.3) was used to present the result of assessment. Each item included in the studies in QUADAS-2 tool was evaluated as yes, no or unclear [21, 22]. The Metavir liver fibrosis staging evaluation system was used. The system was consistent with the liver fibrosis stage of Batts-Ludwing and Scheuer evaluation system, and was divided into five stages of F0, F1, F2, F3 and F4, among which F0 was non-fibrotic. F ≥ 1 was mild liver fibrosis. F ≥ 2 was significant liver fibrosis. F ≥ 3 was severe liver fibrosis. F ≥ 4 was early cirrhosis.

### Statistical analysis

According to the required information, data extraction form was created to calculate relevant indicators, and the data was processed by STATA (version 14.0).

#### Assessment of heterogeneity and publication bias

The Q test was used to evaluate the heterogeneity of the included literatures, and the degree of heterogeneity was determined according to the I^2^ value. If I^2^ ≤ 25%: less heterogeneity, 25% < I^2^ ≤ 50%: moderate heterogeneity, I^2^ ≥ 50%: greater heterogeneity. When the heterogeneity is large, the bivariate mixed effect model should be further adopted. Deeks’ funnel plot was used to detect publication bias when detecting publication bias, and *P* ≤ 0.01 indicated that the publication bias was more significant.

#### Summery statistics

By processing the original data of the included studies, true positive, false positive, false negative and true negative were extracted. Systematic review was performed using STATA 14.0 software for F ≥ 1, F ≥ 2, F ≥ 3, and F ≥ 4 separately in the included literatures, the pooled sensitivity, the pooled specificity, the pooled positive likelihood ratio, the pooled negative likelihood ratio and the pooled diagnostic ratio were calculated. The forest graph and the hierarchical summary receive operating characteristic (HSROC) curve were drawn, and the area under the curve (AUC) was calculated to obtain the AUROC value and its confidence interval. *P* < 0.05 indicates statistical significance.

#### Meta regression and subgroup analysis

If there is a high heterogeneity in the included studies, the single independent regression of the continuous independent variables is performed by STATA 14.0 software, and the sub-combinations of each independent variable are calculated and the results obtained.

#### Sensitivity analysis

In order to observe the stability and heterogeneity of the results of the summery statistics, each included literature was excluded for pooled analysis, and the summery statistics were performed on each group. The results of the summery statistics, I^2^ and those obtained before the exclusion were compared to observe the results.

## Results

### Literature screening results and basic characteristics

**Fig. 1:**
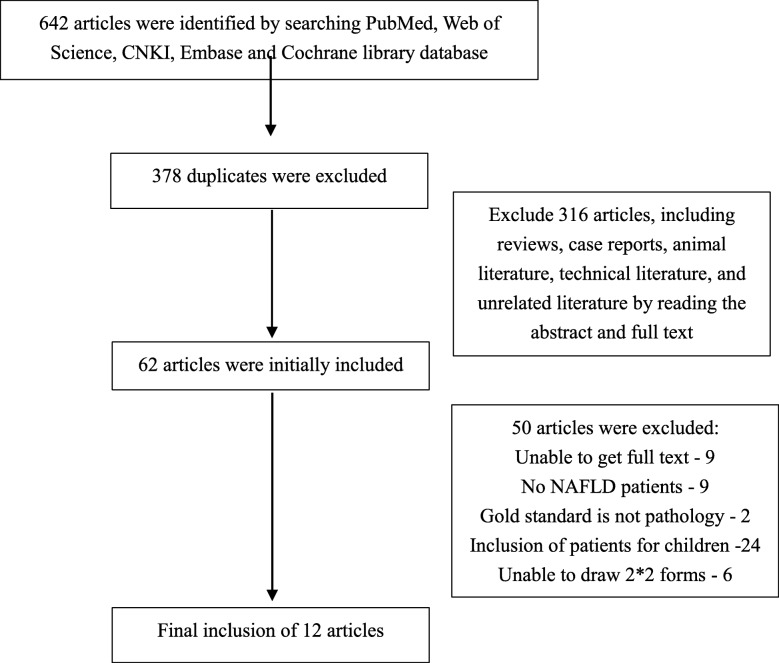
Flow chart for electronic databases search and selection of studies in the systematic review

A total of 642 related studies were preliminarily obtained through the search, and 12 studies [3, 10–20] were finally included for pooled analysis after reading abstracts and full-text screening. A total of 910 patients were included. Of the 12 included articles, 10 [3, 10–14, 17–20] were prospective studies and 2 [15, 16] were retrospective studies. All the included literatures could extract the data of four grids. The study screening process and results are shown in Fig. 1. The basic characteristics of the patients included in the studies are shown in Table 1. The quality evaluation of the included studies is shown in Table 2.

**Table 1:** Characteristics of the studies included in the systematic review

First author	Year	Study design	Country	Subject	Age (y)	BMI (kg/m^2^)	Field strength	Interval	Scoring system	Comparison
Loomba	2016	prospective	US	100	36.6–63.8	27.1–37.1	3 T	46d	NASH-CRN	3D-MRE vs 2D-MRE
Cui	2016	prospective	US	125	33.5–64.3	24.7–38.7	3 T	46.5d	NASH-CRN	MRE vs ARFI
Loomba	2014	prospective	US	117	36.7–63.5	27.4–37.4	3 T	45d	NASH-CRN	–
Costa-Silva	2017	prospective	Brazil	49	41.3–66.3	27.3–37.1	1.5 T	0.3–7.1 m	NASH-CRN	–
Cui	2015	prospective	US	102	37.3–65.3	26.2–37.2	3 T	90d	NASH-CRN	MRE vs eight CPRs
Park	2017	prospective	US	104	36.2–65.4	25.2–35.6	3 T	42d	NASH-CRN	MRE vs TE
Chen	2011	retrospective	US	58	25–78	21.2–50.6	1.5 T	90d	brunt & kleiner	–
Imajo	2016	retrospective	Japan	142	42.9–72.1	23.47–32.73	3 T	< 6 m	brunt & kleiner	MRE vs TE
Wang	2011	prospective	US	5	20–74		1.5 T	,1y	METAVIR	MRE vs DWI
Godfrey	2012	prospective	UK	8	37.5–60.5		1.5 T		Ishak’s score	MRE vs ^31^P MR spectroscopy
Chen	2017	prospective	France	92	45.4–51.75	38.7–41.8	1.5 T		METAVIR	MRE vs VCTE
Huwart	2008	prospective	France	8	41–67	31–34.6	1.5 T	,2d	METAVIR	MRE vs ultrasound elastography vs APRI

**Table 2:**
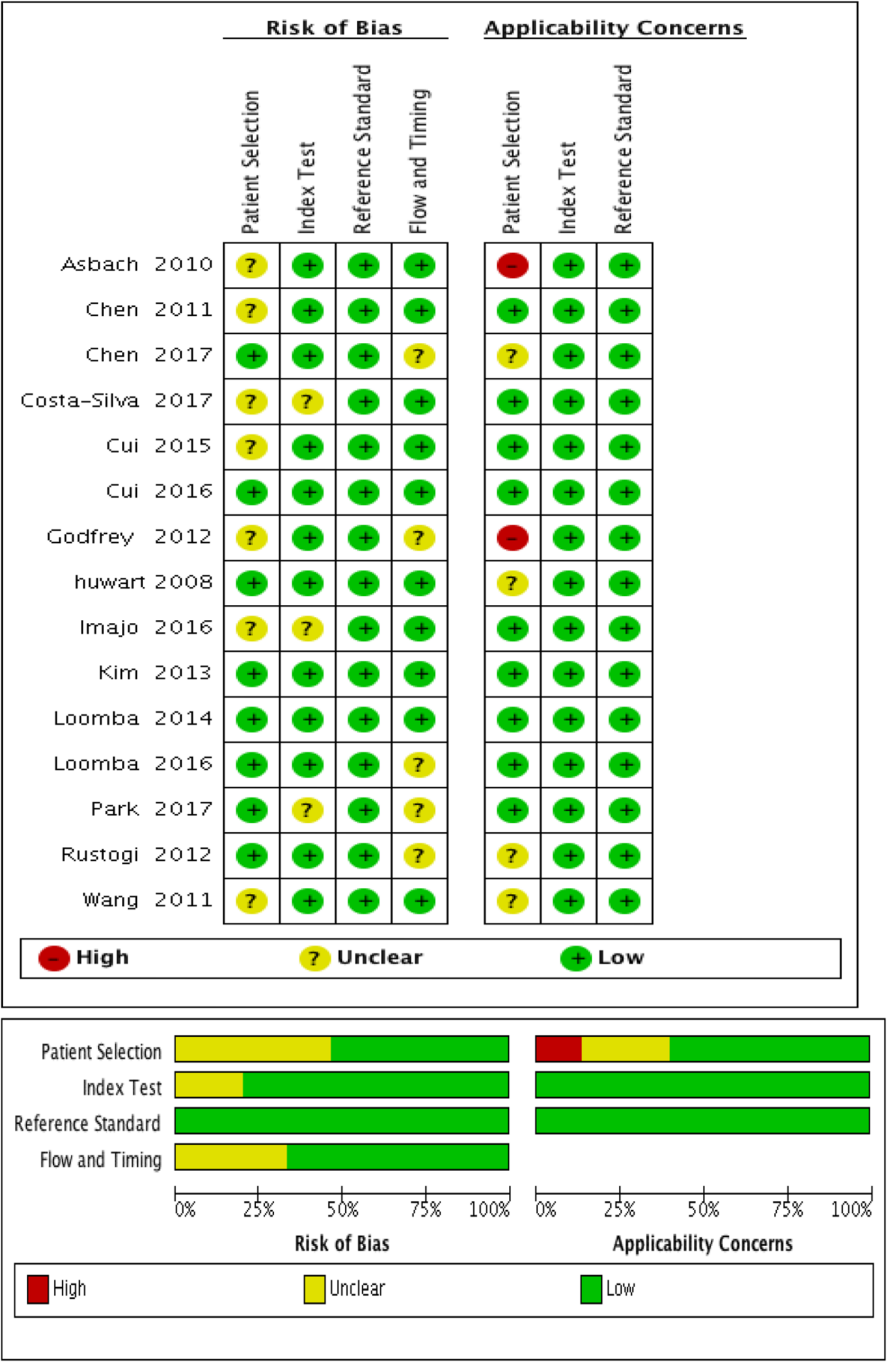
Methodological quality of the 20 included studies

### Pooled analysis results

#### Assessment of heterogeneity and publication bias

**Fig. 2:**
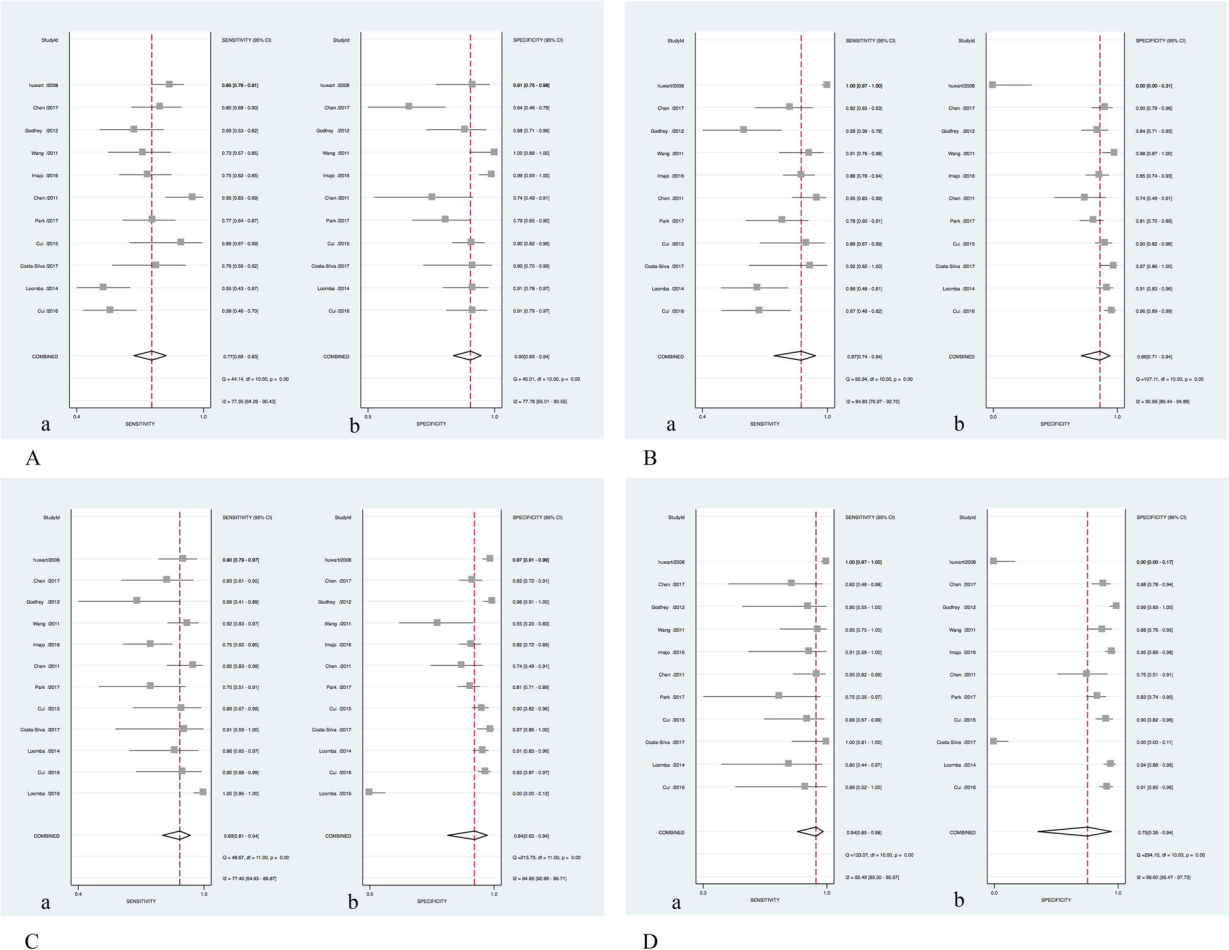
Forest plot of sensitivity and specitivity of MRE for diagnosing different fibrosis stages in NAFLD. A (a): Forest plot of sensitivity for fibrosis stage F ≥ 1, A (b): Forest plot of specitivity for fibrosis stage F ≥ 1; B (a): Forest plot of sensitivity for fibrosis stage F ≥ 2, B (b): Forest plot of specitivity for fibrosis stage F ≥ 2; C (a): Forest plot of sensitivity for fibrosis stage F ≥ 3, C (b): Forest plot of specitivity for fibrosis stage F ≥ 3; D (a): Forest plot of sensitivity for fibrosis stage F ≥ 4, D (b): Forest plot of specitivity for fibrosis stage F ≥ 4

Heterogeneity analysis of F ≥ 1 (mild liver fibrosis), F ≥ 2 (significant liver fibrosis), F ≥ 3 (severe liver fibrosis), and F ≥ 4 (early cirrhosis) groups by Q test. For the sensitivity heterogeneity analysis, the I^2^ values were 77.35, 84.83, 77.40, and 92.49%, respectively. The specific heterogeneity analysis showed I^2^ values of 77.78, 90.66, 94.85, and 96.60%, respectively (see Fig. 2 for details). I^2^ values are greater than 50% suggesting that each group of studies has high heterogeneity and *P* < 0.01, suggesting that the heterogeneity is statistically significant, and a bivariate mixed-effects model is needed for statistical consolidation.

**Fig. 3:**
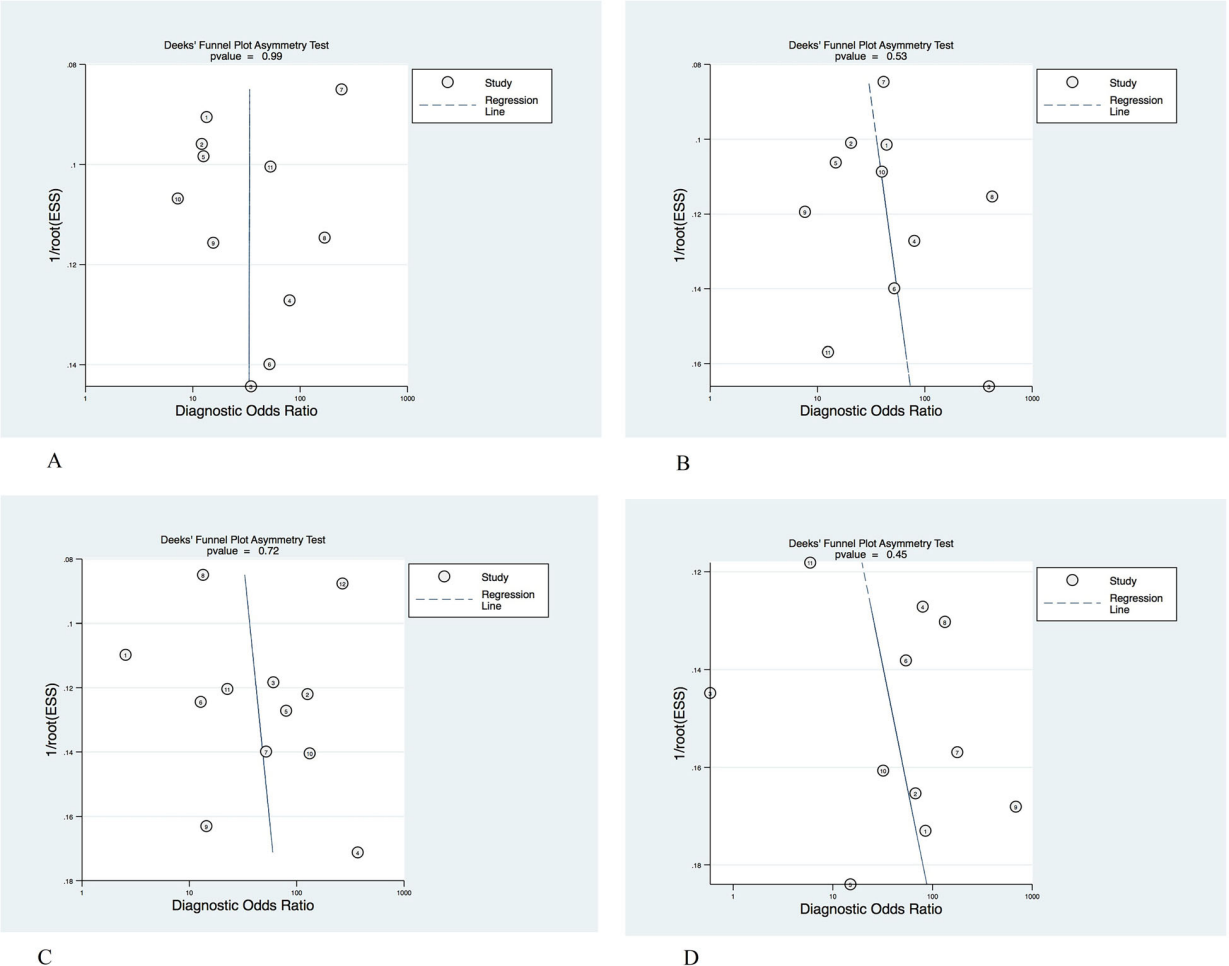
Deeks’ funnel plot indicating the publication bias of the included studies. **a**: Deeks’ funnel plot for fibrosis stage F ≥ 1; **b**: Deeks’ funnel plot for fibrosis stage F ≥ 2; **c**: Deeks’ funnel plot for fibrosis stage F ≥ 3; **d**: Deeks’ funnel plot for fibrosis stage F ≥ 4

The results of Deeks’ funnel plot for F ≥ 1, F ≥ 2, F ≥ 3 and F ≥ 4 groups were shown in Fig. 3. The funnel plot of each group was relatively symmetrical, with *P* values of 0.99, 0.53, 0.72 and 0.45, respectively, showing no statistical significance (*P* > 0.01), indicating that there was no significant publication bias in each group.

#### Summery statistics

**Fig. 4:**
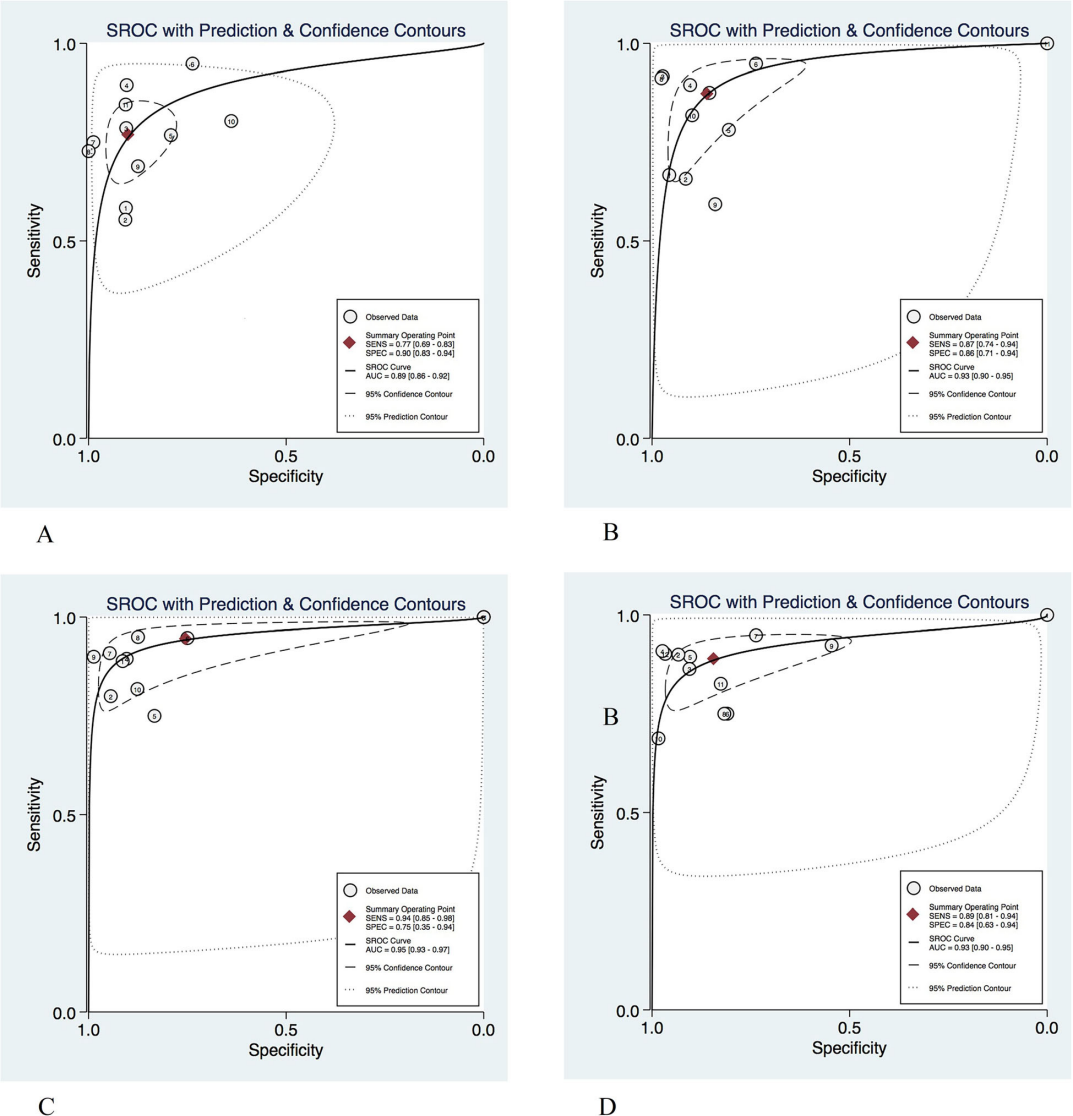
The HSROC curve of MRE for diagnosing different fibrosis stages. The observed data represents the data of each included study; the summary operating point represents the summary sensitivity and specificity; the 95% confidence contour represents the 95% confidence interval of the summary sensitivity and specificity; the 95% prediction contour represents 95% confidence interval of sensitivity and specificity of each individual study included in the meta-analysis. **a**: HSROC curve for fibrosis stage F ≥ 1; **b**: HSROC curve for fibrosis stage F ≥ 2; **c**: HSROC curve for fibrosis stage F ≥ 3; **d**: HSROC curve for fibrosis stage F ≥ 4

By combining the diagnostic indicators of liver fibrosis, the pooled values of sensitivity, specificity, positive likelihood ratio (+LR), negative likelihood ratio (−LR) and diagnostic odds ratio (DOR) were obtained, and the forest graph and HSROC curve were drawn to calculate the AUROC of each group. The forest graph showed that the pooled sensitivity and specificity of each group were 0.77 (95%CI 0.69–0.83) and 0.90 (95%CI 0.83–0.94) respectively, for F ≥ 1; 0.87 (95%CI 0.74–0.94) and 0.86 (95%CI 0.71–0.94) for F ≥ 2; 0.89 (95%CI 0.81–0.94) and 0.84 (95%CI 0.63–0.94) for F ≥ 3; 0.94 (95%CI 0.85–0.98) and 0.75 (95%CI 0.35–0.94) for F ≥ 4, respectively. The area under the SROC curve were 0.89, 0.93, 0.93, and 0.95, respectively. Other details were shown in Table 3. The HSROC curves of the four groups were shown in Fig. 4.

**Table 3:** Pooled analysis results of systematic review for each group, based on 910 patients studies

Stage	Sensitivity (95%CI)	Specitivity (95%CI)	Positive LR (95%CI)	Negative LR (95%CI)	DOR (95%CI)	AUROC (95%CI)	Q test, I^2^		*P* value
							Sen	Spe	
F ≥ 1	0.77(0.69–0.83)	0.90(0.83–0.94)	7.5(4.4–12.7)	0.26(0.20–0.35)	29(15–53)	0.89(0.86–0.92)	77.35%	77.78%	0.99
F ≥ 2	0.87(0.74–0.94)	0.86(0.71–0.94)	6.2(3.0–12.6)	0.15(0.08–0.29)	41(21–81)	0.93(0.90–0.95)	84.83%	90.66%	0.53
F ≥ 3	0.89(0.81–0.94)	0.84(0.63–0.94)	5.6(2.2–14.2)	0.13(0.09–0.21)	42(17–100)	0.93(0.90–0.95)	77.40%	94.85%	0.72
F ≥ 4	0.94(0.85–0.98)	0.75(0.35–0.94)	3.8(1.1–13.1)	0.08(0.04–0.16)	50(16–152)	0.95(0.93–0.97)	92.49%	96.60%	0.45

*LR* Likelihood ratio, *DOR* Diagnostic odds ratio, *AUROC* Area under the receiver operating characteristic

#### Meta regression and subgroup analysis

**Fig. 5:**
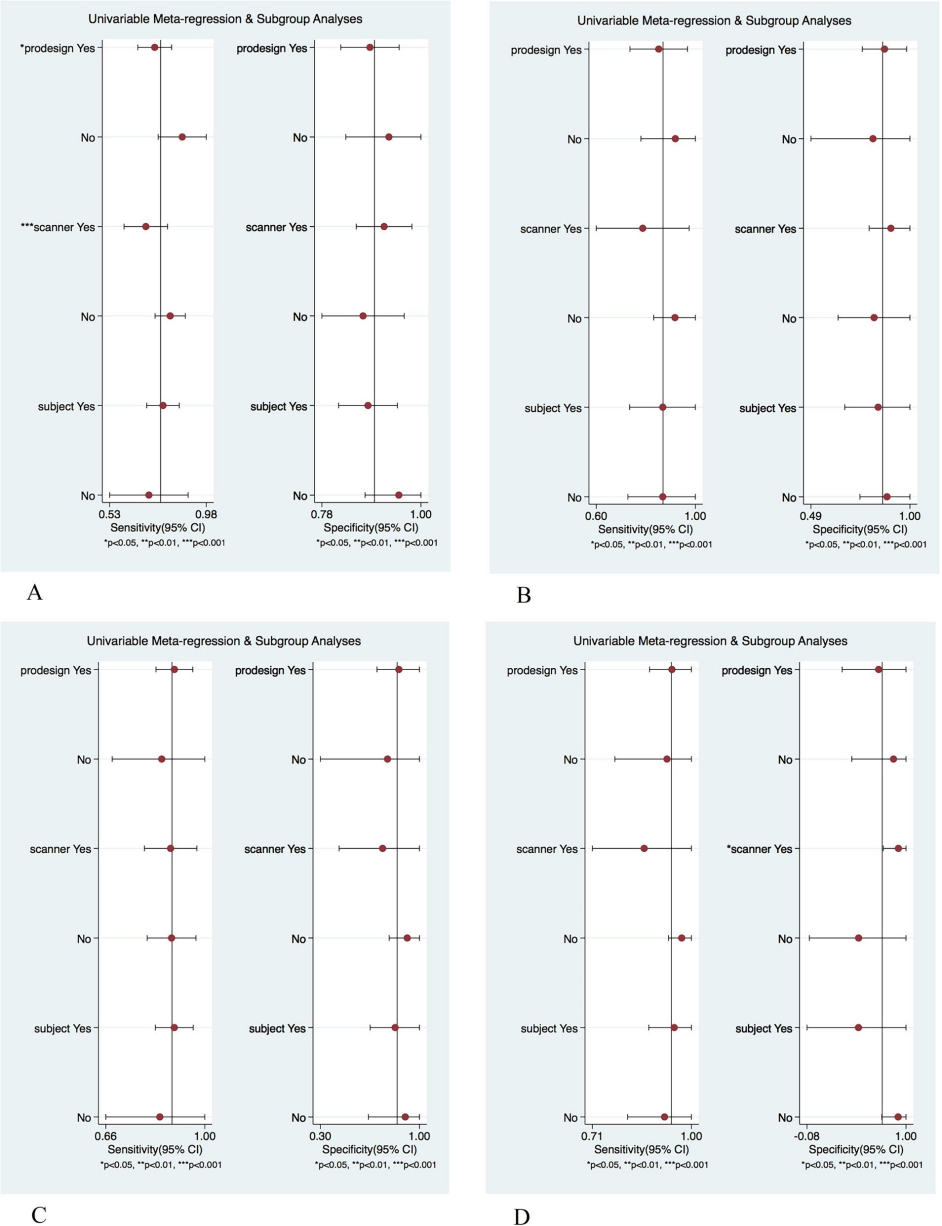
Regression and subgroup analysis results in different fibrosis stages. **a**: The regression and subgroup analysis results of sensitivity and specitivity for fibrosis stage F ≥ 1; **b**: The regression and subgroup analysis results of sensitivity and specitivity for fibrosis stage F ≥ 2; **c**: The regression and subgroup analysis results of sensitivity and specitivity for fibrosis stage F ≥ 3; **d**: The regression and subgroup analysis results of sensitivity and specitivity for fibrosis stage F ≥ 4

In view of the high heterogeneity of the included studies, this paper conducted meta-regression and sub-group analysis. The independent variables of study type (whether prospective or not), equipment field strength (1.5 T or 3.0 T), and the integrity of basic information of subjects (gender, BMI, age) may be the factors leading to high heterogeneity, as shown in Fig. 5.

F ≥ 1 group: The independent variable type (prodesign) and equipment field (scanner) were significant for the heterogeneity of the pooled sensitivity (*P* < 0.05). The independent variables were not statistically significant for the pooled specificity.

F ≥ 2 group: The independent variables had no statistical significance for the heterogeneity of the pooled sensitivity and specificity.

F ≥ 3 group: The independent variables had no statistical significance for the heterogeneity of the pooled sensitivity and specificity.

F ≥ 4 group: The independent device field strength (scanner) was significant for the heterogeneity of the pooled specificity (*P* < 0.05). The independent variables were not statistically significant for the pooled sensitivity.

#### Sensitivity analysis

By separately excluding the results obtained after the inclusion of the study, there were two studies that reduced the heterogeneity of the combined statistic obtained after exclusion. The I^2^ value obtained by the specific heterogeneity analysis was reduced when Loomba 2014 alone was excluded, and the I^2^ value obtained for the sensitivity heterogeneity analysis was reduced when Chen 2017 alone was excluded. The I^2^ obtained after two separate exclusions of the literature was still greater than 50%, but the degree of decline was greater than 10%, indicating that partial inclusion of the literature may be a source of heterogeneity.

## Discussion

In this systematic review, we performed pooled analysis of diagnostic performance of MRE in 12 studies with 910 patients with NAFLD. Regarding the overall diagnostic accuracy of MRE in patients with NAFLD, we found that the pooled sensitivities of MRE for diagnosis of liver fibrosis stage F ≥ 1, F ≥ 2, F ≥ 3, F ≥ 4 were 0.77, 0.87, 0.89, and 0.94 respectively, and the pooled specificities were 0.90, 0.86, 0.84, and 0.75, respectively. The area under the SROC curve was 0.89, 0.93, 0.93, and 0.95, among which MRE has the highest accuracy in F4 stage. Due to NAFLED-associated fibrosis is a strong predictor of mortality, cirrhosis, and hepatocellular carcinoma in patients, it is possible to further develop effective clinical treatment by measuring liver elasticity and staging of liver fibrosis in patients with MRE. And the pooled AUROC of stage F ≥ 2, F ≥ 3 and F ≥ 4was greater than 90%, suggesting excellent discriminative ability for detection of liver fibrosis stage. The optimal threshold values of F ≥ 1, F ≥ 2, F ≥ 3, and F ≥ 4 were different (F ≥ 1 group: 1.77–5.02Kpa, F ≥ 2 group: 2.38–5.37Kpa, F ≥ 3 group: 2.43–5.97Kpa, F ≥ 4 group: 2.74–6.7Kpa), and thus this study couldn’t determine the optimal threshold of fibrosis stage. The possible reasons are as follows: different study designs (10 prospective studies, 2 retrospective studies); different pathological interval time; different MRE technology, parameter setting (6 1.5 T MR, 6 3.0 T MR) and operator’s technical experience; when post-processing is used to obtain an elastic diagram, the region of interest needs to be manually drawn.

In a systematic review by Singh et al. that included 9 studies reporting on 232 patients [5], the pooled AUROC for MRE diagnosis of NAFLD liver fibrosis stage (F ≥ 1, F ≥ 2, F ≥ 3, F ≥ 4) was 0.86, 0.87, 0.90, and 0.91 respectively, and the values of each group are lower than that in our study. The possible reason may arise from 1) we included more included studies in this study; 2) the bivariate mixed effect model was adopted; 3) as a systematic review, the study of Singh et al. is more like a data-sorted analysis without relevant heterogeneity analysis and publication bias analysis. In the meta-analysis by Xiao et al. [23]., they compared the performance of different noninvasive methods for diagnosing liver fibrosis including APRI, FIB-4, BARD score, NAFLD fibrosis score, FibroScan, shear wave elastography (SWE) and MRE in NAFLD, and found that MRE and SWE may have the highest diagnostic accuracy for staging fibrosis in NAFLD patients (the summary AUROC values was 0.96 and 0.95), which accordingly indicated the high accuracy of MRE diagnosis of NAFLD liver fibrosis. However, the study by Xiao et al. is a meta-analysis of multiple examinations applied to NAFLD, the number of relevant studies is relatively small compared with our study, so the specific analysis of MRE is not detailed enough, and the analysis method is less, so the AUC value is lower than this study. In our study, we performed more deep analysis on diagnostic accuracy of the MRE in liver fibrosis stage of NAFLD, further conducted meta regression, subgroup analysis to find sources of heterogeneity, and sensitivity analysis to assess the influence of each study on the overall result. It was proved by many studies that liver stiffness is positively correlated with the severity of liver fibrosis. Elastography is a dynamic imaging technique for measuring the mechanical properties of tissues, which can detect the tissue elasticity (stiffness) [24]. This feature prompts the obvious advantages MRE in the diagnosis of liver fibrosis stage of NAFLD [2, 4].

Georges et al. concluded that hepatocyte swelling and stromal edema (ballooning) caused by inflammation would lead to increased liver stiffness, while Ichikawa et al. observed that hepatitis activity grade may also influence liver stiffness measured using MRE [25, 26]. Therefore, large-scale prospective studies are still needed to investigate the effect of inflammation on the liver hardness measured by MRE. The diagnostic performance is various based on the different technique or machine for MR evaluation. Wagner et al. indicated that the failure rate of overall examination in NAFLD patients was about 7.7%, of which the failure rate of liver MRE was only 3.5% at 3.0 T, while the failure rate was 15.3% at 1.5 T [27]. Loomba et al. performed a prospective study included 100 patients with NAFLD to assess the accuracy of 2D-MRE at 60 Hz, 3D-MRE at 40 Hz and 60 Hz in diagnosing advanced fibrosis [10]. They found that at a threshold of 2.43 kPa, 3D-MRE at 40 Hz had sensitivity 1.0 and specificity 0.94 for diagnosing advanced fibrosis, and concluded that 3D MRE at 40 Hz has the highest diagnostic accuracy in diagnosing NAFLD advanced fibrosis [10]. Compared to 2D-MRE, 3D-MRE allows for improved assessment of spatial patterns of hepatic fibrosis and focal lesions [10]. Salomone et al. suggest that NAFLD fibrosis score can be considered an accurate tool for the stratification of the risk of death in NAFLD patients [28]. Although the diagnostic accuracy of MRE is high as showed by results, it would be important in the future studies to assess the predictive value of MRE for mortality, the “real” outcome for patients with NAFLD.

Due to the high heterogeneity of the included studies, the bivariate mixed effect model was adopted to perform pooled analysis of diagnostic indicators of each group. The sources of heterogeneity were analyzed through sensitivity analysis, meta regression and subgroup analysis and we conclude that the heterogeneity might be related to variety in designs or quality of the included studies, study population and difference in field strength and parameters of MRE equipment. This systematic review has certain limitations: (1) 3 included studies contained other chronic liver diseases, and the amout of NAFLD cases was relatively small relatively to other included studies, and the data may have confound bias, (2) We only retrieves the study in English database, but didn’t search the study published in other languages, which may have information bias, (3) Fibrosis staging errors often occur in clinical diagnosis, so there is the possibility of information bias [29].

## Conclusions

In conclusion, MRE has the advantages of low failure rate, high repeatability, and facilitating biopsy procedures, which can be performed in all subjects without MRI contraindications [30]. At the present, MRE has gradually developed from the scientific research to clinical application, and this systematic review displayed that MRE has a high accuracy in diagnosing the stage of NAFLD liver fibrosis. In the future, the MRE technology should be further optimized to achieve more accurate staging of liver fibrosis in patients with NAFLD, and to provide imaging basis for treatment options and disease prognosis.

## Data Availability

The datasets used and/or analysed during the current study available from the corresponding author on reasonable request.

## Abbreviations

MRE: Magnetic resonance elastography
NAFLD: Non-alcoholic fatty liver disease
QUADAS-2: Quality Assessment of Diagnostic Accuracy Studies
AUROC: Area under the receiver operating characteristic curve
SROC: Summary receiver operating characteristic
+LR: Positive likelihood ratio
-LR: Negative likelihood ratio
DOR: Diagnostic odds ratio
SFL: Simple fatty liver
NASH: Nonalcoholic fatty hepatitis
HCC: Hepatocellular carcinoma
ELF: Enhanced Liver Fibrosis
ARFI: Acoustic radiation force impulse imaging
BMI: Body Mass Index
TP: True positive
FP: False positive
FN: False negative
TN: True negative
HSROC: Hierarchical summary receive operating characteristic
AUC: Area under the curve
SWE: Shear wave elastography
